# mHealth Low-Carbohydrate Type 2 Diabetes Intervention Positively Impacts Sleep Quality and Psychosocial Outcomes

**DOI:** 10.1007/s12529-026-10447-5

**Published:** 2026-03-06

**Authors:** Despina Kolivas, Liz Fraser, Ronald Schweitzer, Peter Brukner, George Moschonis

**Affiliations:** 1 School of Allied Health, Human Services & Sport, La Trobe University - Bundoora Campus, Melbourne, Australia; 2 Watson General Practice, Canberra, Australia; 3 East Bentleigh Medical Group, Bentleigh East, Australia; 4 Department of General Practice, School of Public and Preventative Medicine, Monash University, Melbourne, Australia; 5 Department of Nutrition & Dietetics, Harokopio University of Athens, Athens, Greece

**Keywords:** Type 2 diabetes mellitus, Low carbohydrate, Self-management, MHealth, Online

## Abstract

**Background:**

To examine the 3-month change of a mHealth low-carbohydrate dietary (LCD) application (app) on sleep quality and psychosocial outcomes in people living with type 2 diabetes (T2D). The aim is to understand the broader impact of the LCD app in the Australian primary care context as an adjunct to standard clinical management of T2D.

**Methods:**

The single arm pre-post study recruited community-based people living with T2D, with web access from around Australia, referred via registered supporting general practitioners (GPs). Following informed consent, participants obtained access to the Defeat Diabetes app, which provided education and resources on the LCD approach and support for ongoing management of T2D. Participants self-reported dietary data and validated questionnaires assessed sleep quality (B-PSQI), quality of life (QoL) (EQ-5D-5L), diabetes-related distress (PAID-5), and self-efficacy in diabetes self-management (PDSMS). Univariate regression models examined changes from baseline to 3 months.

**Results:**

The present study included 99 participants (mean age 59 ± 11 years, 55 females). Mean carbohydrate intake reduced from baseline to 3 months as a proportion of overall energy intake (−14%kJ/day, 95% CI −17 to −11). Self-reported perceived health status (6, 95% CI 1 to 11), self-efficacy (5, 95% CI 4 to 7), and diabetes-related distress scores (−2.0, 95% CI −3 to −1) improved over 3 months, and there were nonsignificant improvements in sleep quality.

**Conclusion:**

People with T2D who used a mHealth LCD app significantly improved their perceived health status, diabetes-related distress, and self-efficacy in diabetes self-management scores after 3 months.

**Practice Implications:**

mHealth LCD apps should be considered useful adjuncts to current medical management of T2D.

**Supplementary Information:**

The online version contains supplementary material available at 10.1007/s12529-026-10447-5.

## Introduction

Type 2 diabetes (T2D) poses significant health risks, but it is also widely acknowledged that with early detection and management, T2D is largely preventable and in some cases can be reversed or put into remission [1, 2]. Recent evidence from clinical trials shows that specifically tailored nutritional interventions for T2D, when utilised in conjunction with usual care, are more effective in achieving glycaemic control than usual care alone [3].

In addition, research evidence shows that therapeutic carbohydrate reduction (TCR) for management of T2D improves glycaemic control, reduces the need for medications, assists with weight loss, and aids improvement in blood lipid profiles [4–8]. TCR accommodates both a low-carbohydrate dietary approach (LCD), defined as one in which less than 26% of energy is derived from carbohydrate (or less than 130 g per day), or a very low-carbohydrate ketogenic diet (VLCKD) with less than 10% of energy derived from carbohydrates (between 20 and 50 g of carbohydrate per day) [5, 9].

To implement a lifestyle intervention at scale in a primary care setting, understanding how to best undertake intensive lifestyle changes is required, and best practice management guidelines recommend screening patients to identify relevant psychosocial factors that can affect people’s ability to self-manage their T2D [10, 11]. While there are many factors outside the direct influence of the health care team that can impact patient outcomes, providing counselling, support, and information resources to people with T2D to help improve their self-efficacy has been associated with better glycaemic control and can benefit health-related QoL [12]. Patient counselling and support remain the cornerstone to helping people with T2D understand and manage their condition [13]. In recent years, digital technology and applications provide an adjunct treatment modality for use by both patients and health care professionals alike [14]. Both in-person and technology-mediated peer support can help in improving clinical and psychosocial outcomes in people with T2D. Although there is limited research in this area, a recent review found that the incorporation of peer support mechanisms, such as online support groups, should be recommended by health care providers to people with T2D [15].

Diabetes-related distress is primarily important among these psychosocial factors that can impact self-management [10, 16]. Diabetes distress is distinct from other psychological conditions and is a normal emotional response to the burden of diabetes. It is associated with the ongoing demands of diabetes management such as monitoring blood glucose, taking medication, managing food intake, and physical activity, including thoughts around disease progression. Reduced ability to self-manage, due to diabetes-related distress, is associated with worsening glycaemic control [16, 17]. It is estimated that between 18 and 45% of people living with T2D are affected by diabetes-related distress that may have a negative impact on treatment adherence [10, 18]. In addition, the prevalence of comorbid depression in patients with T2D is estimated to be almost one in four adults, which, if left untreated, can also directly impact on a person’s ability to self-manage [19].

Sleep is another factor that can impact diabetes’ clinical outcomes, and research shows that poor sleep quality and reduced sleep efficiency are correlated with worse glycaemic control in people with T2D [20, 21]. A recent review found that sleep disorders, such as insomnia (34–44%), obstructive sleep apnea (OSA) (55–86%), and restless leg syndrome (8–45%), are more prevalent in people with T2D compared to the general population [22]. In addition, analysis of data from the UK Biobank cohort study in people with T2D taking metformin found a direct correlation linking poor sleep quality, specifically snoring, a symptom of OSA, with HbA1c ≥ 7%, indicative of poor glycaemic control [23]. A lower intake of dietary carbohydrates, through TCR, may have an impact on psychosocial outcomes and in particular sleep quality. A recent cross-sectional study showed that women with T2D who were in the lowest quartile of dietary carbohydrate intake had 69% lower risk of poor sleep quality and 73% lower risk of anxiety compared to those in the highest quartile [24].

In terms of the primary care context, the use of LCD for management of T2D incorporates a multidisciplinary approach that can be resource intensive in providing education and efficient support, and currently almost exclusively undertaken by private health groups and individual primary care physicians [25–28]. While there is an abundance of research evidence supporting the use of LCD interventions for glycaemic control and the management of other clinical outcomes, there is limited research evidence about the impact of LCD digital interventions on sleep quality and psychosocial outcomes in people with T2D [29].

To the best of our knowledge, an experimental study design using the existing health care framework for monitoring, in combination with the use of a LCD app for education and peer support of patients with T2D, has not previously been investigated in the context of the Australian primary care system. The scope of our research is to understand the effect of a digital app in helping people with T2D self-manage their condition specifically examining sleep and psychosocial outcomes, with changes to clinical outcomes including glycaemic control being reported elsewhere [30]. The aim of the current study is to examine the changes in sleep quality, perceived health status, diabetes-related distress, and self-efficacy in the self-management of T2D within the Australian primary care context, in people who used the Defeat Diabetes app over the first 3 months of the intervention.

## Materials and Methods

### Study Design and Participants

Details of the study methods have been published previously [31]. This single-arm pre-post study with a 3-month follow-up is part of a larger study over 12 months. Participant data was collected over the period from October 2022 to May 2024.

As medical monitoring of participants is required for the duration of the intervention, only participants referred via registered supporting GPs were eligible to participate. The study was advertised via GP networks, and GPs who were interested in providing support registered with the research team, who provided detailed study information and patient handouts to assist with recruitment. The primary inclusion criteria were a confirmed diagnosis of T2D with HbA1c ≥ 6.5%, access to a smartphone or PC, and ability to utilise digital apps. Major inclusion/exclusion criteria are outlined elsewhere [31].

All people deemed eligible to participate provided their informed consent for inclusion before participation in the study. Data collection was facilitated by online Research electronic data capture (REDCap) case report forms (CRFs) sent via email to GPs and participants [32, 33].

Approval to conduct the study was granted by the La Trobe University Human Research Ethics Committee (HREC) (approval no. HEC22117). The trial was registered with the Australian New Zealand Clinical Trials Registry (ANZCTR) on 17/05/2022, with the ACTRN: 12622000710729p.

### Study Intervention

After informed consent and baseline data were obtained, participants were granted access to the Defeat Diabetes app and instructed to follow the guidelines over the course of the next 12 months.

The Defeat Diabetes app is a subscription-based commercial app for download on a smartphone (Android and Apple iOS) or use in a web browser and provides a guided educational program on carbohydrate reduction and lifestyle interventions to manage T2D (https://www.defeatdiabetes.com.au/) [34].

On registration confirmation, the participants were sent a series of emails from Defeat Diabetes explaining how to use the app. They are instructed to follow the video lessons in a sequence and use the associated resources that facilitate further understanding of each particular lesson. The Defeat Diabetes app provides low-carbohydrate recipes and cooking demonstration videos, meal planning, shopping lists, and exercise plans, as well as a comprehensive recommended food list with a rating system to guide food choices. The Defeat Diabetes app also encourages users to participate in a moderate amount of physical activity to assist with more effective glycaemic control.

Additional support is provided to users with the option to join a private Defeat Diabetes Community Facebook group. App news and events such as the live and recorded webinars are disseminated via a weekly email newsletter to users. Further information about the app can be found elsewhere [31].

### Participant Baseline Characteristics

Baseline demographic characteristics included age, sex, country of birth, educational history, and coexisting medical conditions, specifically those that may be related to any of the outcome measures. In addition, the time of diagnosis for T2D was recorded, as the recency of diagnosis may be associated with the likelihood of T2D remission [35, 36]. The number of other people living in the participant’s household was also recorded, as this may provide some background into potential barriers or enablers in the success of the intervention. This could take the form of support participants might receive from family members, or the potential barriers to implementation of a LCD, in the context of food preparation and dietary adherence if the participant is also a caregiver.

### Sleep Quality and Psychosocial Outcomes

Participants provided self-reported data on sleep quality, perceived health status, diabetes-related distress, and self-efficacy, which was assessed using validated questionnaires at baseline and after 3 months. A secure weblink to complete the online questionnaires was delivered via email. Detailed information as to the method of distribution of these questionnaires can be found elsewhere [31].

The questionnaires included the Brief Pittsburgh Sleep Quality Index (B-PSQI), which was used to assess sleep quality. The B-PSQI is a six-item questionnaire that incorporates the domains of sleep efficiency, hours of sleep, and sleep latency and can be used to identify problem sleepers. The total score is out of 15, with higher scores indicating worse sleep quality, with a score of greater than 5 indicative of a problem sleeper [37]. QoL was measured using the EQ-5D-5L, a standardised measure of health status that is used to provide a simple generic measure of health status for clinical appraisal and consists of five dimensions, namely mobility, self-care, usual activities, pain/discomfort, and anxiety/depression [38]. In addition, the EQ 5D vertical Visual Analogue Scale (VAS) provides a summary of the participants’ overall current self-perceived health on a scale from 0 to 100, where 0 is labelled ‘The worst health you can imagine’ and 100 is labelled ‘The best health you can imagine’. This scale provides a quantitative measure of the participant’s perception of their overall health.

Two specific diabetes-related questionnaires were also included. The first was the short form Problem Areas in Diabetes Scale (PAID-5), which is a reliable and validated five-item questionnaire that was used to assess diabetes-related distress [39]. A score greater than or equal to 8 in the PAID-5 scale indicates possible diabetes-related emotional distress, with higher scores of up to 20 being indicative of even higher distress. The Perceived Diabetes Self-Management Scale (PDSMS) is an eight-item questionnaire, which was used to assess self-efficacy in the self-management of diabetes. The total score of PDSMS can range from 8 to 40, with higher scores indicating greater confidence in diabetes self-management [40].

### Dietary Intake and Adherence to the Intervention

Three-day food records were completed by participants and submitted via the online REDCap data collection submission forms, via email or via text message to the research team. Data from the food records was entered into FoodWorks Professional 10, Brisbane, Queensland, Australia (Version 10.0.4266) (2020) and a dietary analysis was obtained at baseline and after 3 months [41]. Where a participant was unable or unwilling to complete a 3-day food record, the research team provided the option of a 24-hour dietary recall over the phone, following a standardised process by a trained nutritionist. The dietary data served to elucidate the changes in carbohydrate intake and adherence to the intervention.

### Impact of Physical Activity

Physical activity levels were monitored using the short version of the International Physical Activity Questionnaire (IPAQ) [42], which records physical activity levels during the last 7 days. The IPAQ was completed by participants in a self-administered format. The classifications for physical activity as reported by the IPAQ include high, moderate, and low. A high level of physical activity equates to more than 1 h of moderate intensity physical activity per day. A moderate level of physical activity equates to some activity (about half an hour) of at least moderate intensity physical activity on most days. A low level of physical activity implies that the above criteria are not met.

### Program Evaluation—Application of the Intervention

After 3 months, a program evaluation questionnaire was sent to participants to verify the implementation of the intervention and their experience of the Defeat Diabetes Program, to understand the degree to which participants engaged with the intervention.

### Statistical Analyses

The study design was powered to investigate changes in glycaemic control as measured by the primary outcome HbA1c, which is detailed in the study protocol. The following analysis of data examining sleep and psychosocial outcomes is deemed to be exploratory in nature [31]. Continuous variables were examined for the normality of their distribution using the Kolmogorov–Smirnov test. Univariate linear regression models were used to assess within-group changes in all continuous study outcomes from baseline to the 3-month follow-up. These regression models were adjusted for appropriate covariates, which included sex, age, time since diabetes diagnosis, co-existing medical conditions, and dietary carbohydrate intake. Changes in categorical variables from baseline to the 3-month follow-up were tested using the Chi-square test. To understand the relationship between the changes in sleep and psychosocial outcomes and changes in clinical outcomes, we performed linear regression analyses and calculated the standardised beta coefficient. The statistical analyses were conducted for the total sample, but after also stratifying by sex.

All statistical analyses were performed using SPSS statistical software for Windows (Version 28.0, Armonk, NY). All reported *p* values were two-tailed, and the level of statistical significance was *p* < 0.05.

## Results

For purposes of recruitment and at the 3-month follow-up, a total of 99 participants provided data as per the study protocol.

**Fig. 1:**
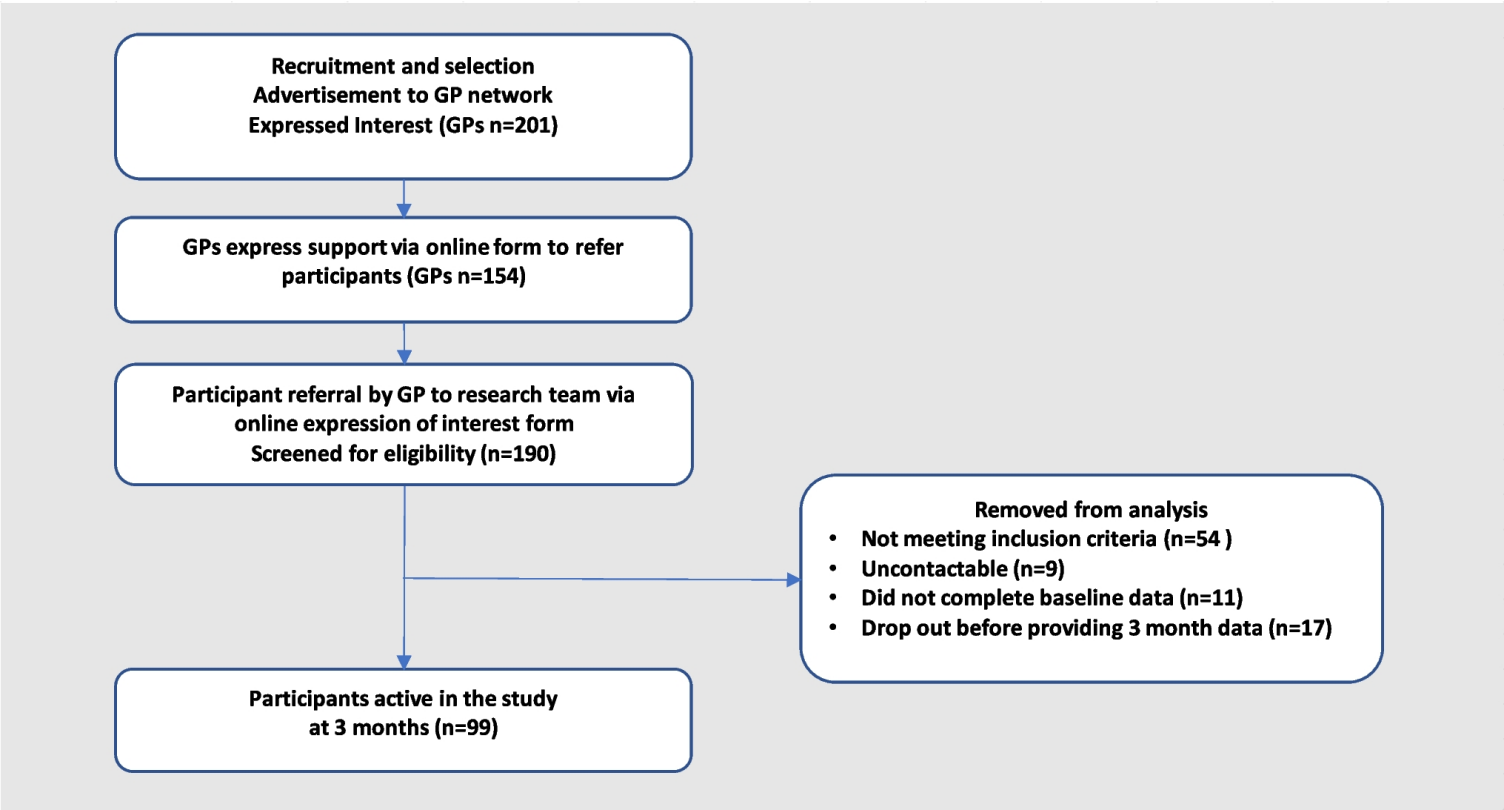
Study process and recruitment

Figure 1 depicts the study flow from recruitment to 3-month follow-up.

### Participant Baseline Characteristics

Table 1 presents the descriptive characteristics of study participants in terms of their socio-demographics, in the total sample (*n* = 99) and by sex. The median time since diabetes diagnosis was 2.9 years, with approximately 30% of participants diagnosed within the last year and others having diabetes for more than 20 years. Seventy-eight per cent of study participants reported having one or more co-existing medical conditions. These included cardiac issues (15%), gastrointestinal disorders (5%), hyperthyroidism or metabolic bone disease (1%), osteoporosis (5%), rheumatoid arthritis (4%), psychological disorders (9%), hypertension (57%), high blood cholesterol (42%), prior gastric bypass surgery (2%), significant kidney or liver disease (4%), and immunodeficiency (2%). According to the classifications as specified by the International Physical Activity Questionnaire (IPAQ), approximately 41% of participants were moderately active, while around 26% and 32% were classified as having low or high levels of physical activity respectively [42].

**Table 1:** Descriptive characteristics of study participants at baseline in the total sample and by sex

	Total sample (***n*** = 99)	Male (***n*** = 44)	Female (***n*** = 55)	***p*** value
Socio-demographics				
Age (years) (mean (SD))	58.4 (11.3)	57.6 (11.2)	59.1 (11.4)	0.52
Education level, ***n*** (%)				0.64
Up to secondary	34.3	31.8	36.4	
Higher education	65.7	68.2	63.6	
Country of birth,*** n*** (%)				0.13
Australia	60.6	52.3	67.3	
Overseas	39.4	47.7	32.7	
Employment status (%)				0.03
Unemployed	5.1	0	9.3	
Casual/part-time/full-time	63.3	75.0	53.7*	
Retired	31.6	25.0	37.0*	
People in household (%)				0.64
One person	18.2	16.3	20.0	
2 or more people	80.8	83.7	80.0	
Years with type 2 diabetes (median (IQR))	2.9 (6.0)	2.0 (5.1)	4.0 (7.0)	0.02
Time since diagnosis (%)				0.01
Up to 6 years	68.7	79.5	60.0	
6 or more years	31.3	20.5	40.0	
Co-existing medical conditions (%)				0.39
None	22.2	18.2	25.5	
One or more	77.8	81.8	74.5	
Diabetes medications (%)				0.48
No medication	28.3	31.8	25.5	
Medications	71.7	68.2	74.5	
Antihypertensive medications (%)				0.86
No medication	46.5	45.5	47.3	
Medications	53.5	54.5	52.7	
IPAQ activity level (%)				0.50
Low	26.3	20.5	30.9	
Medium	41.4	45.5	38.2	
High	32.3	34.1	30.9	

*p *values that compare continuous variables between sexes are derived from the independent sample *T*-test or the nonparametric Mann–Whitney test, i.e. as per the normality of their distribution. The *p* values that compare categorical variables are derived from the chi-square test. *p* < 0.05 in the pairwise comparisons in proportions between sexes

*Statistically significant pairwise differences between males and females

### Changes in Dietary Intake

After 3 months of intervention there was a significant decrease in average total energy intake (−1570 kJ/day, 95% CI: −2128 to −1013). This change was observed in both males (−1757 kJ/day, 95% CI: −2648 to −867) and females (−1416 kJ/day, 95% CI: −2122 to −710).

**Fig. 2:**
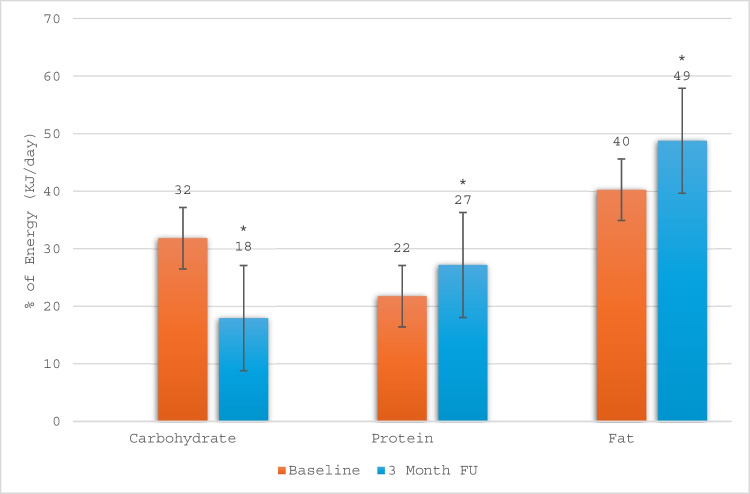
Average dietary macronutrient intake distribution as a proportion of total energy intake for the total sample at baseline and after 3 months. The asterisk indicates the statistically significant difference between baseline and 3-month follow-up (*p* < 0.05). Error bars depict standard error. Contribution to energy intake from alcohol, fibre, and other nutrients is excluded from this analysis

Participants’ macronutrients’ intake expressed as proportion of energy intake changed significantly from baseline to the 3-month follow-up, as illustrated in Fig. 2. A significant reduction in dietary carbohydrate intake (−14%kJ/day, 95% CI −17 to −11) was recorded, while significant increases were observed in dietary protein (6%kJ/day, 95% CI 4 to 7) and total fat (9%kJ/day, 95% CI 6 to 11) intakes. These significant changes were observed in both males and females. Fourteen participants completed 24-hour dietary recalls in lieu of 3-day food records, and three participants did not provide any food record data at 3 months.

### Changes in Physical Activity Levels

After 3 months of intervention, there were no significant differences in the percentage of participants allocated in the low, medium, and high activity physical activity level categories in the total sample and when stratified by sex (data not presented in tables).

### Sleep Quality

After 3 months, there were no significant changes observed in the overall B-PSQI sleep quality score as well as in subjective sleep quality (assessed by the question, “How would you rate your sleep quality overall?”) in the total sample (supplementary figure S1) and when the analyses were stratified by sex. Even though there were no statistically significant changes in the overall B-PSQI sleep quality score and subjective sleep quality, the number of problem sleepers (i.e. those identified as having a score of greater than 5) decreased from 59% at baseline to 51% in 3 months (supplementary figure S2).

### Quality of Life

After 3 months of intervention, there were no significant differences from baseline in each of the five dimensions of the EQ-5D-5L. However, there were more participants reporting that they experienced no issues in each specific dimension (i.e. mobility, activities of daily living, personal care, pain/discomfort, anxiety/depression) after 3 months of intervention, most notably in the anxiety/depression dimension. Supplementary figure S3 (a-e) shows a graphical representation of the changes in each dimension over time.

Table 2 shows the change in self-perceived health status (EQ-5D VAS) in the total sample and as part of stratified analyses. After 3 months, there was a significant improvement in the EQ-5D VAS in the total sample (6, 95% CI 1 to 11). This was largely driven by females (9, 95% CI 3 to 16) with no significant change found in males. Significant improvements were also seen in those who had a higher education level (7, 95% CI 1 to 13), were born in Australia (7, 95% CI 0 to 13), had T2D for less than 6 years (7, 95% CI 1 to 13), were employed (8, 95% CI 2 to 14), were moderately active (7, 95% CI 0 to 14), and were able to reduce their dietary intake of carbohydrates to less than 26% of total energy intake (6, 95% CI 0 to 11).

**Table 2:** Changes in self perceived health status (EQ-5D VAS) from baseline to 3 months of intervention in the total sample and subgroups

	Baseline			Follow-up			3-month change			
	***n***	Mean	SD	***n***	Mean	SD	Mean change	(95% CI) Lower	(95% CI) Upper	***p*** value
EQ-5D VAS										
Total sample	99	64	19	97	70	17	6	1	11	0.02
Male	44	67	19	42	69	19	2	−6	10	0.63
Female	55	62	19	55	71	16	9	3	16	0.01
Education level										
Up to secondary	34	63	18	33	68	19	5	−4	14	0.31
Higher education	65	65	20	64	72	16	7	1	13	0.03
Country of birth										
Overseas	39	65	19	37	70	19	5	−4	13	0.27
Australia	60	64	19	60	71	16	7	0	13	0.04
Employment status										
Unemployed	5	41	31	5	48	17	7	−29	42	0.67
Casual/part-time/full-time	62	63	18	61	71	17	8	2	14	0.01
Retired	31	70	16	30	72	16	2	−7	10	0.71
Years with type 2 diabetes										
< 6 years	68	63	20	68	70	17	7	1	13	0.03
≥ 6 years or more	31	68	18	29	72	19	5	−5	14	0.33
Co-existing medical conditions										
None	22	64	16	21	72	16	7	−3	18	0.15
One or more	77	64	20	76	70	18	6	0	11	0.06
Activity level										
Low	26	53	22	22	60	17	6	−5	17	0.29
Medium	41	66	17	38	70	17	7	0	14	0.05
High	32	72	16	37	77	15	5	−2	13	0.15
Carbohydrate intake as a % of energy at 3 months										
Up to 26% kJ/day	75	65	18	75	71	17	6	0	11	0.04
More than 26% kJ/day	21	64	24	20	69	18	5	−8	17	0.47

*p* values were derived from the independent sample *T*-test and indicate the statistical significance of the changes from baseline to 3-month follow-up

*SD*, standard deviation from the mean; *CI*, confidence interval.

### Diabetes-Related Emotional Distress

Table 3 shows the change in PAID-5 score in the total sample and as part of stratified analyses. After 3 months, PAID-5 score was significantly reduced in the total sample (−2, 95% CI −3 to −1). Both males (−2, 95% CI −4 to 0) and females (−2, 95% CI −3 to 0) reported decreases in PAID-5 scores, indicating an improvement in diabetes-related distress. Significant improvements were also seen in those who had a higher education level (−2, 95% CI −4 to −1), were born in Australia (−2, 95% CI −3 to −1), had T2D for less than 6 years (−2, 95% CI −4 to −1), had one or more co−existing medical conditions (−2, 95% CI −3 to −1), were employed (−2, 95% CI −4 to −1), were classified as moderately active (−3, 95% CI −5 to −1), and were able to reduce their dietary intake of carbohydrate to less than 26% of total energy intake (−2, 95% CI −4 to −1).

**Table 3:** Diabetes-related emotional distress (PAID-5) score for overall sample from baseline to 3 months of intervention in the total sample and subgroups

	Baseline			Follow-up			3-month change			
	***n***	Mean	SD	***n***	Mean	SD	Mean change	(95% CI) Lower	(95% CI) Upper	***p*** value
PAID-5										
Total sample	99	6	4	97	4	4	–2	–3	–1	< 0.001
Male	44	6	4	42	4	4	–2	–4	0	0.03
Female	55	7	4	55	5	4	–2	–3	0	0.01
Education level										
Up to secondary	34	6	4	33	5	4	–2	–4	1	0.15
Higher education	65	6	4	64	4	4	–2	–4	–1	0.01
Country of birth										
Overseas	39	7	4	37	5	5	–2	–4	0	0.07
Australia	60	6	4	60	4	3	–2	–3	–1	0.01
Employment status										
Unemployed	5	8	2	5	6	4	–2	–7	3	0.40
Casual/part-time/full-time	62	7	4	61	5	4	–2	–4	–1	0.01
Retired	31	5	4	30	4	4	–1	–3	1	0.29
Years with type 2 diabetes										
< 6 years	68	7	4	68	5	4	–2	–4	–1	0.01
≥ 6 years or more	31	5	4	29	4	4	–2	–4	0	0.12
Co-existing medical conditions										
None	22	6	4	21	5	3	–2	–4	1	0.14
One or more	77	6	4	76	4	4	–2	–3	–1	0.01
Activity level										
Low	26	8	4	22	7	5	–1	–3	2	0.58
Medium	41	7	5	38	4	3	–3	–5	–1	0.01
High	32	5	3	37	3	4	–2	–3	0	0.06
Carbohydrate intake as a % of energy at 3 months										
Up to 26% kJ/day	75	6	4	75	4	4	–2	–4	–1	0.01
More than 26% kJ/day	21	7	3	20	6	4	–1	–4	1	0.28

*p* values were derived from the independent sample *T*-test and indicate the statistical significance of the changes from baseline to 3-month follow-up

*SD*, standard deviation from the mean; *CI*, confidence interval

The PAID-5 score was divided into categories indicating the level of distress (i.e. those participants who had scores greater than or equal to 8 indicating potential distress and those whose score was below 8 indicating no distress), at baseline and 3 months and shown in supplementary figure S4. A statistically significant difference was found between the groups, with a decrease in the percentage of participants experiencing distress at 3 months (18%) compared to the relevant percentage at baseline (35%) (*p* < 0.05).

### Perceived Diabetes Self-efficacy

After 3 months, PDSMS showed a statistically significant increase in the total sample (5, 95% CI 4 to 7), which was indicative of improvement in participants’ self-efficacy in self-managing their T2D (Table 4).

**Table 4:** Perceived diabetes self-efficacy scores (PDSMS) from baseline to 3 months of intervention in the total sample and subgroups

	Baseline			Follow-up			3-month change			
	***N***	Mean	SD	***n***	Mean	SD	Mean change	(95% CI) Lower	(95% CI) Upper	***p*** value
PDSMS										
Total sample	99	25	6	97	30	6	5	4	7	< 0.001
Male	44	26	6	42	31	5	5	2	7	< 0.001
Female	55	24	6	55	30	7	6	3	8	< 0.001
Education level										
Up to secondary	34	25	6	33	30	7	5	2	8	0.01
Higher education	65	25	6	64	31	5	5	3	7	< 0.001
Country of birth										
Overseas	39	26	7	37	31	5	6	3	9	< 0.001
Australia	60	25	5	60	30	6	5	3	7	< 0.001
Employment status										
Unemployed	5	26	6	5	30	6	4	−6	13	0.37
Casual/part-time/full-time	62	25	5	61	30	5	5	3	7	< 0.001
Retired	31	25	7	30	31	7	5	2	9	0.01
Years with type 2 diabetes										
< 6 years	68	25	5	68	30	6	5	3	7	< 0.001
≥ 6 years or more	31	24	7	29	30	6	6	3	10	< 0.001
Co-existing medical conditions										
None	22	24	6	21	31	6	7	3	10	< 0.001
One or more	77	25	6	76	30	6	5	3	7	< 0.001
Activity level										
Low	26	24	5	22	29	5	5	2	9	< 0.001
Medium	41	26	7	38	29	5	4	1	7	0.01
High	32	25	5	37	32	7	6	3	9	< 0.001
Carbohydrate intake as a % of energy at 3 months										
Up to 26% kJ/day	75	25	6	75	31	5	6	4	8	< 0.001
More than 26% kJ/day	21	25	7	20	26	7	2	−3	6	0.45

*p* values were derived from the independent sample *T*-test and indicate the statistical significance of the changes from baseline to 3-month follow-up

*SD*, standard deviation from the mean; *CI*, confidence interval

Improvement of diabetes self-efficacy scores were seen in both females (6, 95% CI 3 to 8) and males (5, 95% CI 2 to 7). The same applied for all other subgroups, where statistically significant improvements were also observed in diabetes self-efficacy (*p* < 0.05). The only exceptions were those participants who were unable to reduce their dietary intake of carbohydrates to less than 26% of total energy intake and those participants who reported that they were unemployed at baseline.

### Regression Analysis with BMI and HbA1c

It has been previously reported that the intervention led to a significant reduction in the primary outcome HbA1c (−1.0%, 95% CI −1.3 to −0.7) and nonsignificant reduction in BMI (−1.3 kg/m^2^, 95% CI −3 to 0.5) across the study cohort after 3 months of intervention [30]. A regression analysis was performed for each outcome variable to understand the relationship to changes in clinical outcomes BMI and HbA1c in the study cohort. Table 5 shows the linear regression standardised beta coefficient (*β*) for each outcome in the total sample and by sex.

**Table 5:** Regression coefficients examining the associations between the changes in BMI and HbA1c levels with sleep quality and the examined psychosocial outcomes in the total sample and by sex

	ΔBMI (%)		ΔHbA1c (%)	
	β*	***p ***value	***β***†	***p*** value
ΔB-PSQI (%)				
Total sample	0.05	0.67	0.214	0.07
Males	−0.276	0.12	0.036	0.83
Female	0.049	0.76	0.404	0.02
ΔEQ-5D VAS (%)				
Total sample	−0.042	0.70	−0.303	0.02
Males	0.044	0.80	−0.373	0.03
Female	0.045	0.78	−0.45	0.01
ΔPAID-5 (%)				
Total sample	0.017	0.89	0.017	0.89
Males	−0.125	0.50	−0.072	0.69
Female	0.046	0.90	0.089	0.61
ΔPDSMS (%)				
Total sample	−0.127	0.27	−0.138	0.23
Males	−0.127	0.47	−0.099	0.57
Female	−0.028	0.86	−0.296	0.08

*Β*, standardized beta coefficient

*Adjusted for the ΔHbA1c

^†^Adjusted for ΔBMI

The standardised beta coefficients (*β*) show a significant association between the reduction in HbA1c and the B-PSQI (*β* = 0.404, *p* = 0.02) in female participants, and with self-reported health status as measured by the EQ-5D VAS (*β* =  −0.303, *p* = 0.02) across the cohort. No significant associations were observed in the other outcome variables and BMI.

### Program Evaluation—Participants’ Satisfaction with the Defeat Diabetes App

After 3 months, participants were asked about their experience using the Defeat Diabetes app by completing a program evaluation. Approximately 80% of participants reported a high level of satisfaction and at that time 88% of participants reported that they were currently using the resources provided by the Defeat Diabetes app, and 85% of participants reported that they were planning to continue to use the resources.

In addition, 54% of participants reported that they had access to Facebook. Of this group, 81% of these participants reported using the Defeat Diabetes Members’ community Facebook group at 3 months, with 97% of these participants planning to continue to use the Facebook group.

## Discussion and Conclusion

### Discussion

We conducted a single-arm pre-post study that aimed to examine the effect of an mHealth LCD app on glycaemic control and explore the impact on sleep quality and psychosocial outcomes in people with T2D. As reported elsewhere, we found that there was a significant improvement in HbA1c after 3 months of using the Defeat Diabetes app and, on average, the amount of dietary carbohydrate consumed by study participants decreased significantly as did overall dietary energy intake [30]. The results show that participants compensated for the average reduction in carbohydrate intake by increasing both total fat and protein intake in line with the Defeat Diabetes app recommendations. Increasing levels of protein and fat while lowering the proportion of carbohydrate consumed is likely to result in higher levels of satiety and consequently a reduction in energy intake. We found significant improvements in study participants’ psychosocial outcomes after 3 months of engaging with the app. This was especially notable in self-reported health status (EQ VAS) and diabetes-related distress, and for self-efficacy for the self-management of T2D. Greater improvements were noted in those participants who could reduce their level of dietary carbohydrate intake to 26% or lower as a proportion of their energy intake indicating adherence to the intervention.

Although there were no statistically significant improvements in sleep quality in the cohort, there was a nonsignificant improvement in subjective sleep quality scores and a decrease in the number of participants classified as problem sleepers at 3 months. Better sleep quality can play a large role in improving quality of life and consequently may lead to better health outcomes [43]. We found a significant correlation between reductions in HbA1c with sleep quality scores in female participants. 24 In women with obesity, it has been shown that adherence to a LCD improved sleep quality with a proposed mechanism of action being reduction in fat mass and/or subsequent improvement in inflammatory markers [44, 45]. It should also be noted that sleep issues are multifactorial and the prevalence of poor sleep quality can be on average as common in those with and without T2D [46].

A significant improvement was seen in participants’ self-reported health status as indicated by the increase observed in the total EQ-5D VAS score after 3 months. Research evidence on self-management and education interventions shows improvement in glycaemic control in people with T2D [47]. Reported QoL improvements were found to be dependent on the type and mode of delivery of the intervention, with web interventions being found to be equally as effective as traditional modes of delivery [48, 49]. In this regard, studies examining web-based program focusing on self-management and facilitation of behaviour change showed promising results in both clinical markers and improving QoL [50]. Our regression analysis also showed improvement in self-reported health status across the cohort, as per the EQ-5D VAS, was associated with improvements in HbA1c. Although physical activity has been proposed as another lifestyle measure for improving physical and mental health outcomes and QoL, our study showed no significant changes in the percentages of participants categorised with different levels of physical activity at baseline and 3 months follow-up [51]. This could indicate that the improvements in perceived health status observed in the current study were directly attributable to LCD and the subsequent reduction in HbA1c.

In 2016, the American Diabetes Association issued a position statement to address specific psychosocial outcomes in people with diabetes, including recommendations for screening of diabetes-related distress and mental health conditions [10]. It is acknowledged that these factors can make it more difficult for people with T2D to achieve their clinical management goals and improvements in mental health status can lead to better self-management [52]. A digital continuous remote care intervention that focused on carbohydrate restriction found that depressive symptoms improved and were maintained over a 2-year period and were correlated with the degree of adherence to low-carbohydrate eating [53]. In this regard, although our results showed non-significant improvement in the depression/anxiety dimension of the EQ-5D-5L QoL questionnaire, the improvements in the relevant score may be of clinical significance, since they are indicative of better mental health status amongst participants after 3 months of intervention. In addition, there was a significant improvement in diabetes-related distress as measured by the PAID-5 questionnaire across the entire study cohort. It has been established that diabetes-related distress is a mediator of glycaemic control separate to major depressive disorder or depressive symptoms [17, 54]. A recent study conducted in a primary care setting in the UK, utilising a web-based diabetes self-education intervention, reported reductions in HbA1c and diabetes-related distress with greater improvements in those most recently diagnosed with T2D [48, 55]. In addition, our results also support that a LCD can reduce diabetes-related distress scores in people with T2D, and aligns with an Australian study that found improvement in diabetes-related distress scores over a 12-month period [56]. It is important to highlight that our regression analysis did not find a significant correlation between the PAID-5 score and HbA1c in our study participants.

Improvements were also seen in participants’ perceived self-efficacy for the self-management of their T2D. Prior research has found that education-focused interventions can increase diabetes’ knowledge and promote self-management behaviours that can lead to significant improvements in glycaemic control and other health outcomes [47, 57]. Recent international studies in outpatient clinics that incorporated a web-based diabetes self-education program found statistically significant improvements in the intervention group’s HbA1c as well as in PDSMS scores, compared to the control group over the course of a 6-month period [58, 59]. From our results, it can be inferred that the improvement in PDSMS score in our study cohort is most likely due to nutrition education resources provided to study participants through the app. The nutrition education resources included video training lessons, detailed articles, low-carbohydrate recipes, and meal plans, with most participants (85%) reporting that they plan to continue to use the resources after 3 months. It is notable that perceived self-efficacy improved at all levels of physical activity, indicating that the intervention has applicability for people who may have limited mobility or are inactive.

Our exploratory regression analysis also showed significant associations between the changes in certain psychosocial factors and the changes in HbA1c over the 3-month follow-up period. More specifically, the decreases observed in HbA1c were found to be significantly associated with improved sleep quality scores in female participants. The negative association observed between HbA1c and the EQ-5D VAS across the whole sample indicates improvement in self-perceived health status with reduction in HbA1c as discussed earlier [30].

#### Strengths and Limitations

A key strength of the methodology is the use of validated questionnaires for the assessment of sleep and psychosocial outcomes. These questionnaires were conveniently delivered in an online format, via email and weblink to minimise reporting burden to participants; however, it should be noted that two participants did not complete the questionnaires at the time of reporting. The compromise to reduce reporting burden by utilising the short form versions of the Pittsburgh Sleep Quality Index (PSQI) and the Problem Areas In Diabetes (PAID) questionnaires may have impacted the precision in detecting changes [37, 39].

An additional strength of the current study was the use of 3-day food records (and as an option 24-hour dietary recalls) to obtain an accurate assessment of dietary intake. Further clarification was sought directly from participants to get a better understanding of the dietary data recorded if required. This allowed participants to complete the food records at a convenient time with minimal reporting burden. However, there was also a limitation in this aspect, since from the food record data submitted at baseline, it was apparent that a small group of participants had begun to decrease their intake of carbohydrates after referral but before providing informed consent to participate, due to their eagerness to commence a low-carbohydrate eating approach. It should be noted that the current dietary guidelines for Australia and New Zealand recommend an acceptable macronutrient distribution range (AMDR) of between 15 and 25% of energy from protein, 20 and 35% of energy from fat, and 45 and 65% of energy from carbohydrate for healthy children and adults [60]. Further limitations regarding the use of food records in the context of the current study have been described elsewhere [30].

In terms of further limitations, participants were recruited on the understanding that they were able to use digital applications; however, there was no way to assess their competence in the use of digital tools. In addition, not all participants were Facebook users, and as such would not have had access to peer support. Another limitation is the lack of a control group; in this regard, however, the subgroup analysis conducted revealed that participants who had greater adherence to the intervention, which was ascertained by the food record data after 3 months of intervention, had better outcomes. It is important to note that the study was powered to detect a change in the primary outcome HbA1c; as such, the impact of the intervention on the outcomes presented should be considered exploratory in nature. And although validated questionnaires were used in prior T2D research in a global setting, not all of the questionnaires may be fully validated in an Australian context; however, this alone does not exclude their validity more generally.

Within this study cohort, we expect that there would be some degree of inherent selection bias, as these participants are routinely monitored under standard medical guidance, and have an existing relationship with their health care provider. Because of this, it is difficult to elucidate the impact on the self-reported health outcomes of the Hawthorne effect [61].

#### Future Directions

Future research will assess the effects of an mHealth LCD app over a longer period of 12 months. The impact of the intervention could be further investigated by understanding the underlying motivation and readiness for undertaking lifestyle changes, as this is an important feature as to the applicability [62]. New features that can be incorporated into the app design could include psychological support and motivational aspects for lifestyle changes, as these are likely to contribute to adherence, engagement, and sustainability. Future studies could compare a case-matched cohort utilising conventional medical treatment as a control group for the outcomes measured in study participants.

### Conclusion

We aimed to assess the effect of a mHealth LCD app in helping people with T2D improve sleep quality and psychosocial outcomes through education and support that are based on the principles of the LCD approach. Our study showed that in addition to improved glycaemic control reported previously, participants achieved significant improvements in perceived health status, diabetes-related distress, and self-efficacy scores, as well as nonsignificant improvements in subjective sleep quality. Results at 6 and 12 months will enable us to understand longer-term adherence and sustainability.

### Practice Implications

Our research has shown that the overall health status of participants was improved through the use of the app. As such, the use of an mHealth LCD app should be prescribed as an additional tool that health care providers can offer to their patients seeking lifestyle modifications to help manage their T2D.

## Supplementary Information

Below is the link to the electronic supplementary material.

ESM 1 (DOCX 1.85 MB)

## Data Availability

The data sets used and analysed during this study will be available from the corresponding author on reasonable request.
