# Supplementary material for: mHealth Low-Carbohydrate Type 2 Diabetes Intervention Positively Impacts Sleep Quality and Psychosocial Outcomes
Source: Int J Behav Med. 2026 Mar 6;33(2):195–209. doi: 10.1007/s12529-026-10447-5 (PMC13161327; doi:10.1007/s12529-026-10447-5)
Supplement: Supplementary file 1 — (DOCX 1.85 MB) [file 12529_2026_10447_MOESM1_ESM.docx]

**mHealth low-carbohydrate type 2 diabetes intervention positively impacts sleep quality and psychosocial outcomes**

**Supplementary Information**

Figure S1 Subjective sleep quality domain in B-PSQI “How would you rate your sleep quality overall?” at baseline and after 3 months.

Figure S2 Problem sleepers as identified by the B-PQSI score of greater than 5 at baseline and after 3 months.

Figure S3a EQ-5D-5L Mobility dimension at baseline and after 3 months.

Figure S3b EQ-5D-5L Personal Care dimension at baseline and after 3 months.

Figure S3c EQ-5D-5L Usual Activities dimension at baseline and after 3 months.

Figure S3d EQ-5D-5L Pain/Discomfort dimension at baseline and after 3 months.

Figure S3e EQ-5D-5L Anxiety/Depression dimension at baseline and after 3 months.

Figure S4 PAID-5 score, comparing those with no diabetes distress (score <8) and those with distress indicated (score ≥ 8) at baseline and after 3 months.
